# Effects of Resistance Respiratory Training on Respiratory Muscle Strength in Healthy Active Individuals

**DOI:** 10.3390/muscles5020034

**Published:** 2026-05-08

**Authors:** Antonela Karmen Ivišić, Dario Vrdoljak, Nikola Foretić, Vladimir Pavlinović, Ivan Drviš

**Affiliations:** 1 Faculty of Kinesiology, University of Split, 21000 Split, Croatia; antivi@kifst.hr (A.K.I.);; 2 Centre for Heart, Lung and Vascular Health, University of British Columbia, Okanagan Campus, Kelowna, BC V1V 1V7, Canada; 3 Faculty of Kinesiology, University of Zagreb, 10110 Zagreb, Croatia; ivan.drvis@kif.unizg.hr

**Keywords:** forced breathing, exhalation, expiratory pressure, inspiratory pressure, Airofit

## Abstract

Background: Respiratory muscle strength (RMS) is a critical factor influencing athletic performance, particularly in high-intensity or prolonged activities. RMS encompasses inspiratory (IMs) and expiratory muscles (EMs), which differ in anatomical structure, fiber composition, and responsiveness to training. Methods: This pilot interventional within-subject study investigated the effects of two resistive respiratory muscle training (RMT) protocols on RMS and small airway function in eight physically active adults (two females, six males). Maximal inspiratory (MIP) and expiratory pressures (MEP), along with pulmonary function tests (PFTs), were measured using the Airofit PRO™ device and spirometry before and after two consecutive 7-day training protocols, with a 2-day break between interventions. The workload was progressively increased by lengthening the duration of forced inhalation and exhalation, while keeping the air resistance constant. Results: Results demonstrated significant improvements in MEP across both protocols and after a 10-day washout period (*p* < 0.001–0.03), whereas MIP showed no significant changes (*p* = 0.19–0.66). Moderate transient improvements were observed in small airway flow (MEF25%) following the first protocol (ES = 0.62), which regressed after the second. Conclusions: These outcomes suggest differential responsiveness of respiratory muscles to RMT; EMs, characterized by a higher proportion of fast-twitch type II fibers and a predominantly passive role in normal breathing, respond rapidly to short-duration, high-intensity forced expiration training through neuromuscular adaptations. Conversely, IMs, dominated by slow-twitch type I fibers, require longer-duration, higher-load training to elicit meaningful adaptations, explaining the limited changes in MIP. Small airway function appeared minimally trainable due to structural and physiological constraints, with short-term improvements likely reflecting effort-dependent factors rather than lasting adaptations. Finally, RMT can selectively enhance EM performance through appropriately designed short-duration, high-intensity interventions, while IMs may necessitate prolonged or higher-load stimuli. The findings highlight the importance of targeted training strategies, individualized to muscle fiber composition and functional demands, to optimize respiratory performance. Future research should investigate longer interventions, larger diverse cohorts, and precise measurement methods to further elucidate RMT’s effects on both respiratory muscles and small airway function.

## 1. Introduction

The respiratory system, as a physiological parameter, is a key factor in sports performance [1]. This is particularly relevant for athletes engaged in long-duration and high-intensity activities (e.g., running, swimming, cycling, team sports games) [2]. Moreover, respiratory muscle strength (RMS) and endurance play a crucial role in sports characterized by high ventilatory demands involving breath-holding or apnoea (e.g., freediving) [3]. Hence, respiratory muscles must sustain elevated pressures to maintain thoracic stability and effective gas exchange. Also, it is important to distinguish the different roles of respiratory muscle groups engaged in the inspiration and expiration breathing phases [3]. The diaphragm and external intercostal muscles are the primary muscles of inspiration, with accessory muscles contributing during increased ventilatory demand [4]. Yet forceful expiration is performed by the abdominal muscles and internal intercostals, which actively increase intrathoracic pressure to generate expiratory flow [5]. Considering the above, it is necessary to conduct respiratory muscle testing in order to assess physiological condition and identify potential weaknesses.

RMS assessment is commonly conducted using maximum inspiratory (MIP) and expiratory pressures (MEP) that measure the maximal efforts of the respiratory muscles [6]. Furthermore, MIP is a measure related to enhanced inspiratory muscle function, reduced work of breathing, and delayed onset of respiratory muscle fatigue during intense exercise. On the other hand, higher MEP values contribute to more effective expiration and greater ventilatory efficiency at high breathing frequencies [7,8]. Insufficient inspiratory or expiratory muscle strength increases respiratory muscle fatigue and dyspnoea, which can limit exercise tolerance [9]. Moreover, respiratory muscle fatigue may activate the respiratory muscle metaboreflex, reducing blood flow to locomotor muscles and negatively affecting endurance and overall athletic performance [10].

Balloon catheters represent a gold standard for inspiratory and expiratory muscle strength assessment (i.e., MIP and MEP). Specifically, the precise physiological procedure is used to determine the diaphragmatic function and optimal respiratory muscle function for athletic performance. Briefly, oesophageal (Poes) and gastric pressure (Pga) are measured using oesophageal and gastric balloon catheters to directly quantify diaphragm and expiratory muscle execution (Pdi) [11]. However, these invasive techniques require the insertion of catheters through the nose and into the oesophagus and stomach, which is often unpleasant for the subject and may cause discomfort, gagging, nasal irritation, or anxiety [12]. Also, there is a certain limitation in these techniques’ restricted applicability in sport environments. Therefore, MIP and MEP are commonly assessed via portable devices in field-based settings [13,14].

Among mouth-based devices, small and portable Airofit PRO™ is specifically designed for respiratory muscle training (RMT), being easier to operate and providing information on MIP and MEP [15,16]. Due to its adjustable airflow resistance, it enables pressure transmission during maximal respiratory efforts [17]. Additionally, the rigid chamber and controlled leak system (for MIP) reduce pressure artifacts caused by glottic closure or cheek compliance, thereby improving measurement validity compared with other mouth-based devices. As MIP and MEP measured with the Airofit PRO™ device reflect pressures generated against a closed system, they are consistent with societal guidelines for respiratory muscle testing and are comparable to values obtained with laboratory-grade manometers [15]. Therefore, the Airofit PRO™ device is a valid asset in both RMS diagnostics and enhancement. In addition, there is a need to establish different training protocols for different roles of respiratory muscles.

Accordingly, RMS is enhanced through respiratory muscle strength training (RMST) using devices that provide resistance and possible improvement of athletes’ performance [18,19,20]. Nevertheless, previous studies show different outcomes when investigating RMS in rowers. Some authors reported increased values of RMS parameters through RMST with non-significant improvement in athletes’ 2000 m performance [21]. In contrast, Perera, et al. [22] established that RMST improves rowing performance, although it does not significantly enhance RMS or delay fatigue. Despite the growing literature investigating the effect of RMT, there is a lack of studies examining standardized respiratory training regimes for optimal enhancement of specific respiratory muscles, specifically standardized and directly comparable training protocols targeting inspiratory and expiratory muscles separately. Additionally, limited evidence exists on the short-term responsiveness of specific respiratory muscle groups and small airway function to different resistive training interventions, particularly in field-based conditions using portable devices. Therefore, it is important to investigate the practical interventions for and duration of possible muscle adaptation due to the applied training protocol. Therefore, this study aims to investigate possible effects of different resistive respiratory muscle training (RMT) interventions on the function of respiratory muscles.

## 2. Results

**Figure 1:**
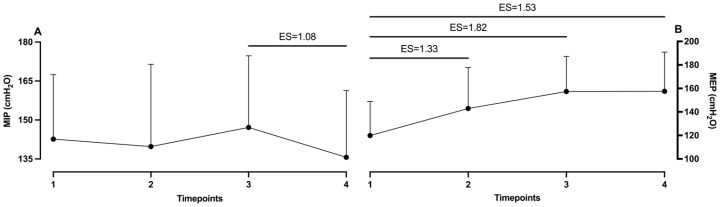
Effect sizes between measurement timepoints in (**A**) MIP and (**B**) MEP. ES, effect size; 1, baseline; 2, after first protocol; 3, after second protocol; 4, after washout.

The differences among measurement timepoints are shown in Table 1. It can be noted that MIP did not show any significant differences among measurements. Oppositely, in the MEP measurement, statistically significant differences were found between the baseline measurement and those after Protocol 1 (*p* = 0.03), after Protocol 2 (*p* < 0.01) and after washout (*p* = 0.01). Observation of effect sizes shows that the third measurement and washout had a moderate ES (1.08) in MIP. In MEP, significant differences obtained by post hoc analysis were defined. Specifically, the baseline measurement showed a large ES with the second (1.33), third (1.82) and washout measurements (1.53) (see Figure 1).

**Figure 2:**
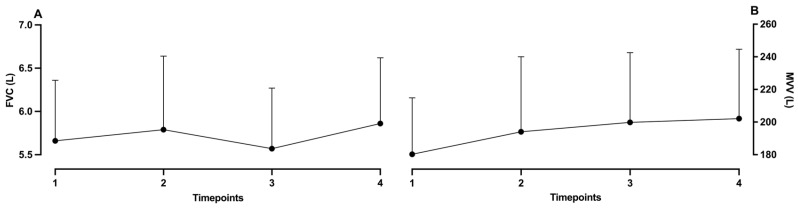
Effect sizes between measurement timepoints in (**A**) FVC and (**B**) MVV; 1, baseline; 2, after first protocol; 3, after second protocol; 4, after washout.

**Figure 3:**
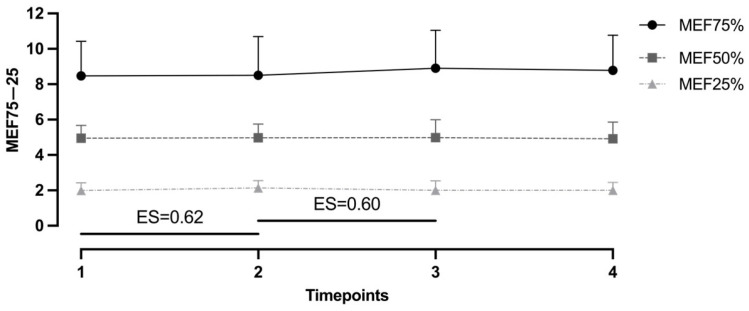
Effect sizes between measurement timepoints in FEF75–25%. ES, effect size; 1, baseline; 2, after first protocol; 3, after second protocol; 4, after washout.

Observation of differences among all PFT variables showed no statistical significance (see Table 2 and Figure 2). However, when including ES, the analysis showed improvement from baseline to the first measurement in MEF25% with a moderate effect size (0.62). Similarly, moderate improvement can be noted between the second and third measurements (ES = 0.60) (see Figure 3).

## 3. Discussion

This study aims to investigate possible effects of resistive RMT on the function of respiratory muscles. The study results showed several important findings: (1) the four-week respiratory training program induces a significant change in MEP but not in MIP measurements, (2) training effects on MEP were evident 10 days after the washout period, and (3) a moderate improvement in small airway flow—particularly in MEF25%—was observed following the first training regimen.

### 3.1. Differences in Training Response: Inspiratory vs. Expiratory Muscles

Research on the effects of RMT on respiratory muscle strength has predominantly been conducted in patient and athletic populations. Given that our sample consisted of physically active individuals, the authors believe that the discussion should align more closely with studies involving athletes. However, findings from studies examining the impact of RMT on RMS in athletic populations have been largely inconsistent. In a systematic review of HajGhanbari et al. (2013) [19], the authors found that IMT/RMT participants had greater improvements in MIP than the control, and this effect differed among sports. Greater improvement in MIP was demonstrated for cycling, endurance track sports, intermittent sprint-type sports, and rowing, whereas swimmers, divers, and special forces athletes showed no significant differences. Subgroup analyses of athlete level demonstrated that both elite and recreational athletes who performed RMT had greater improvements in MIP than the control group [19]. Yet, according to Sperlich et al. (2009), the resistive-type trainer, like we used in our study, did not significantly improve MIP compared with control group values [23].

Specificity of RMT is mostly connected with training regimen. Following the rules for peripheral muscle training adaptations, training with high force and low velocity increases maximal force whereas training with high velocity and low force increases maximal muscle shortening velocity [24]. Since respiratory flow is proportional to the velocity of muscle shortening and respiratory pressure is proportional to force generation it would be logical to expect that training that emphasizes velocity would increase maximal respiratory flow while training that emphasizes force would increase maximal respiratory pressure. Most probably this is a major reason why the subjects in our study experienced increases in MEP but not in MIP measures, specifically after changes in the training protocol in the 3rd and 4th weeks. Since most of the subjects did not have any experience with respiratory muscle training, the training program for the first two weeks was designed to adapt to breathing patterns and increase thorax flexibility to avoid any possible injuries caused by RMT, and to increase lung compliance during the forced breathing process. Furthermore, the 3rd and 4th weeks were more invasive and involved more resistance and longer loading intervals. The greater resistance and longer workload obviously activated more expiratory muscles. This can be explained by the time duration in which these muscles were involved. Namely, in the second part of the study we increased the forced breathing time, which led to a significant overall increase in the workload on the expiratory muscles. It is well known that controlled forced expiration is longer in duration than forced inspiration, in ratios of approximately 1:2–1:4 [25]. When workload time was increased in the second part of the study for 10 s per interval it caused significantly more expiratory than inspiratory muscle engagement. Consequently, this was shown as a significant increment in MEP but not in MIP.

### 3.2. Respiratory Muscles: IMs’ and EMs’ Response to RMT

The study results demonstrated significant improvement in MEP testing, with a persisting training effect 10 days after the washout period. Such a finding could be explained by several factors, including anatomy indices and inherited physiological features of respiratory muscles, together with distinctive responsiveness to training stimuli between inspiratory (IMs) and expiratory muscles (EMs).

When it comes to physiological observation, it is essential to highlight certain anatomy features, including muscle fiber types, and the different physiological functions of respiratory muscles. Due to their continues role in regular everyday breathing, IMs, especially the diaphragm, have a high level of endurance in healthy individuals [26]. Moreover, the diaphragm is characterized by a high percentage of type I (slow-twitch) oxidative fibers, with a high fatigue resistance ratio and greater adaptation to endurance [27,28]. Therefore, these muscles require higher-load stimuli for increasing adaptation. In contrast, EMs (e.g., abdominal muscles, internal intercostal muscles, external oblique muscles) do not have an active role in regular breathing, since expiration is mainly the passive phase of breathing [29]. Nevertheless, previous studies demonstrated that singers have higher levels of activation of EMs when compared to the general population, which indicates a strength increase in EMs with forced expiration training [30,31]. Notably, EMs have a lower proportion of type I fibers, which may indicate that these muscles have lower endurance, are more responsive to repeated, forced expiration training, and are prone to fatigue during heavy exercise [29,32].

Different muscles require specific training stimuli because of the differences in the physiological features of their fiber structures. Previously, authors demonstrated that type I oxidative fibers are predominantly involved in prolonged aerobic activities [33,34]. Specifically, these muscle fibers have a higher fatigue threshold and lower force production compared to type II [35,36]. Therefore, type I muscle fibers are more responsive to long-duration, low-intensity training [37,38]. Oppositely, type II (fast-twitch) muscle fibers exhibit distinctly different physiological characteristics. Wilson, Loenneke, Jo, Wilson, Zourdos and Kim [34] noted that types IIA and IIX facilitate short-duration anaerobic activities and are proportionally higher in elite power and strength athletes. Furthermore, Grgic, Homolak, Mikulic, Botella and Schoenfeld [37] demonstrated that type II fibers possess superior force-generating capabilities (e.g., faster calcium kinetics, faster shortening velocities) compared to type I oxidative fibers. As aforementioned, the training responsiveness of both type I and II muscle fibers is fundamentally determined by their distinctive physiological differences. Due to their higher fatigue resistance, type I muscle fibers require greater time under training loads to stimulate accentuated growth [37]. On the other hand, resistance training loads (≥90% 1 RM) are preferential in terms of hypertrophy of type II fibers, considering their power-generating ability [39]. Thus, respiratory muscle training should be customized considering the specific physiological characteristics of IMs and EMs.

Taken together, these physiological distinctions help explain the observed sustained improvement in MEP after the 10-day washout period following respiratory muscle training. Furthermore, the present findings may be attributed to several interrelated physiological and neuromuscular mechanisms, specifically concerning the capabilities of EMs. Additionally, considering their higher percentage of type II fibers, EMs exhibit rapid responsiveness to short-duration and high-intensity training stimuli. As previously stated, these fibers have the ability to generate greater force and adapt primarily through neuromuscular mechanisms (i.e., motor unit recruitment and firing frequency), which may occur early in the training process and can be sustained for several days, even without consistent stimuli [40,41]. As EMs have a passive role during regular breathing, they are predominantly activated during forced expiration (i.e., voluntary contractions). Therefore, we may assume that training load applied through RET represents a relatively strong and novel stimulus for these muscles. Consequently, this may lead to neuromuscular adaptation in short training periods. Furthermore, enhanced voluntary activation and motor coordination of EMs, which are underutilized during regular breathing, contribute to improvements that persist even after the 10-day washout period. To corroborate this, previous studies demonstrated that a short term without training (i.e., ≤2 weeks) typically does not result in significant declines in neuromuscular performance, particularly when initial improvements are mainly the result of neuromuscular changes rather than structural adaptations [41,42,43]. Overall, the sustained enhancement in MEP following the 10-day washout period in the present study may be the result of several factors, including early-phase neuromuscular adaptations, increased efficiency of EM recruitment, and the inherently fast-adaptive profile of the type II fibers predominant in these muscles.

### 3.3. Changes in Small Airway Performance After Different Training Regimens

Small airway function was assessed using MEF25% (maximal expiratory flow at 25% of FVC), a parameter reflecting airflow during the terminal phase of forced exhalation and commonly used as an indirect indicator of peripheral airway function [44,45]. Although small airways are structurally distinct—characterized by the absence of cartilaginous support and low baseline resistance [46,47]—their functional assessment via spirometry remains challenging due to the high variability and effort dependency of flow-based indices [48,49]. The results of our study demonstrated a moderate improvement in MEF25% following the first training regime, which was not sustained after the second protocol. From a physiological perspective, this pattern likely reflects improvements in expiratory muscle coordination and effort rather than true adaptation of the small airways. MEF parameters are derived from the maximal expiratory flow achieved at specific lung volumes and are therefore highly sensitive to the force and technique of expiration [44,50]. Consequently, the shorter and less demanding initial training protocol may have facilitated optimal performance during testing, while the more intensive second protocol may have introduced fatigue, reducing expiratory efficiency.

This interpretation is supported by previous research indicating that flow-dependent spirometric indices, such as MEF25%, are highly variable and influenced by effort, limiting their reliability as markers of physiological adaptation [48]. Similarly, Lin et al. (2002) highlight that transient functional changes in muscle performance may not translate into sustained physiological adaptations, particularly when underlying structural characteristics constrain adaptability [36]. In the context of small airways, their limited smooth muscle content and structural properties suggest a reduced capacity for functional training-induced changes, with adaptations being predominantly structural and thus unlikely to occur over short intervention periods [46,50].

When comparing our findings with previous respiratory muscle training (RMT) studies, a consistent pattern emerges. While RMT has been shown to improve respiratory muscle strength and global pulmonary function, its effects on small airway indices remain inconsistent. For example, Chen et al. (2024) reported improvements in mid-expiratory flow (FEF25–75) only after prolonged training (24 weeks), suggesting that longer interventions may be necessary to influence peripheral airway function [51]. In contrast, shorter-duration interventions, such as those applied in our study, appear insufficient to induce lasting changes. Furthermore, systematic reviews and meta-analyses demonstrate that RMT primarily enhances parameters such as maximal inspiratory and expiratory pressures, with limited or inconsistent effects on flow-dependent spirometric indices [52,53]. The decline in MEF25% values toward baseline following the second training protocol in our study may also be explained by fatigue-related mechanisms. Increased training intensity does not necessarily translate into improved spirometric outcomes and may, in some cases, attenuate performance due to reduced neuromuscular efficiency [54]. This aligns with broader evidence suggesting that small airway parameters exhibit low trainability and high intra-individual variability [49].

Overall, our findings are consistent with the existing literature indicating that MEF25% is a highly effort-dependent parameter with limited sensitivity to short-term training adaptations. The absence of sustained improvement in our study likely reflects the combined influence of measurement variability, fatigue, and the inherently low adaptability of small airway structures, rather than the ineffectiveness of the training protocols themselves.

## 4. Materials and Methods

### 4.1. Participants

In this study, 8 healthy active participants were included (2 females and 6 males), with a mean chronological age of 35.55 years, body mass of 78.05 kg, and body height of 180.01 cm. No participants with clinical complications were included in the study. All participants taking part in this study volunteered and were informed about the purpose of the study. Experimental procedures were completed following the Declaration of Helsinki, and they were approved by the corresponding authors’ institutional research ethics board (Ethics Board Approval 2181-205-02-05-24-007).

### 4.2. Variables

The variables included in this study are anthropometric indices, the pulmonary function test (PFT), and maximal expiratory/inspiratory pressures (MIP and MEP). Anthropometric indices included body height, body mass, and body fat percentage. Body mass and body fat percentage were assessed with a bioimpedance scale (Tanita BC 418 scale; Tokyo, Japan). Body height was determined with a Tanita HR-001 anthropometer (Tanita; Tokyo, Japan). The maximal pressures of respiratory muscles were analyzed using a portable mouth pressure manometer device (AirOFit PRO™, Copenhagen, Denmark, company https://www.airofit.com/, accessed on 5 May 2026). Pressure analysis included maximal inspiratory and maximal expiratory pressures (MIP and MEP) measured using the device. Both MIP and MEP are assessed via a mouthpiece against an occluded airway, representing the maximal pressures generated during inspiration and expiration, respectively [54].

PFT was done using computerized spirometry (Quark PFT, Cosmed, Rome, Italy). Pulmonary function, including forced vital capacity (FVC), maximal expiratory flow at 75%, 50%, and 25% of the forced vital capacity (MEF75, MEF50, MEF25), and maximal voluntary ventilation (MVV), is assessed by spirometry using forced breathing maneuvers. FVC represents the maximal volume of air exhaled forcefully after full inspiration, MEF values indicate expiratory flow rates at 75%, 50%, and 25% of FVC, and MVV is measured as rapid, deep breathing performed over approximately 12–15 s and extrapolated to one minute [55].

### 4.3. Procedure

**Figure 4:**
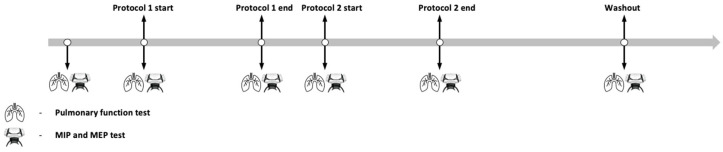
Study timeline showing measurement timepoints and protocol durations with breaks between.

During this study, all participants completed two different training protocols. Protocol 1 consisted of separate inspiratory and expiratory training blocks, while Protocol 2 used combined intervals with simultaneous forced breathing and an emphasis on reaching total lung capacity (TLC). The aim was to compare the efficacy of different resistive respiratory muscle training (RMT) modalities and their effects on respiratory muscle function. Before starting with training, PFT and MIP/MEP were tested to obtain baseline values. Both protocols included breathing training with an interchange of expiration and inspiration. The protocols were performed over a 7-day period (10 min per day), with a 2-day break between them to allow adequate recovery from training and to minimize potential training fatigue and motivational decline (i.e., prevent participant habituation or reduced engagement) (see Figure 4). Participants were doing the breathing protocols with a respiratory muscle trainer constructed from natural rubber that measures 11.25 cm in length, with a mouthpiece width of 5.5 cm. The total weight of the device is 0.06 kg. The lower section of the device contains a valve, 3 cm in width, through which air flows during inhalation and exhalation. By rotating the valve to the left, the airflow passage is reduced, thereby increasing breathing resistance. Conversely, rotating the valve to the right enlarges the airflow passage and decreases breathing resistance. The valve can be adjusted to six distinct positions: position 1 corresponds to the lowest airflow (i.e., the highest breathing resistance), while position 6 allows the highest airflow (i.e., the lowest breathing resistance). Airflow resistance is identical during both inhalation and exhalation. Participants were instructed to maintain the valve at position 3 throughout the experimental procedure. The workload was progressively increased by extending the duration of forced inhalation and exhalation.

Protocol 1 was done with 2 min of regular breathing in the pressure regulation device. Succeeding that, 3 min were spent focusing on inspiration. During these 3 min, participants performed 30 s of forced inspiration with 30 s of normal breathing. After this interval, the next 3 min focused on expiration, with 30 s of forced expiration and 30 s of normal breathing. To finish the protocol, they performed 2 min of regular breathing. The total time of each training session was 10 min.

Protocol 2 was done with 1 min of regular breathing in the pressure regulation device. Following that, 3 intervals were done, with every interval combining 40 s of forced expiration and forced inspiration, followed by 20 s of regular breathing. Then, 1 min of regular breathing for the recovery period was included, followed by 3 more intervals in the same manner as the first. To finish the protocol, participants performed 2 min of regular breathing. In addition, subjects were instructed to continue inhaling after the forced respiration until total lung capacity (TLC) was reached. The total time of each training session was 10 min. Following the 16-day RMT intervention, participants underwent a washout period. This period was an interval without RMT training stimuli with the primary purpose of assessing the duration for which the effects of the training program persisted.

Instructions regarding the RMT protocol and its key features were delivered in person by the principal investigator to each participant individually. This procedure was conducted prior to each protocol, i.e., twice in total. Participants were allowed to contact the study coordinator at any time during the RMT program if needed. Given that all participants were experienced exercisers, the need for additional intervention or clarification during the program was minimal.

### 4.4. Statistical Analysis

The Shapiro–Wilk test was used to assess the normality of the distribution, and confirmed that all variables were normally distributed. To determine the differences between measurement timepoints, repeated measures ANOVA was done with the Fisher LSD test. The *p* values in all analyses were tested with the magnitude-based Cohen’s effect size (ES) statistic with modified qualitative descriptors (trivial ES < 0.2; small ES = 0.21–0.60; moderate ES = 0.61–1.20; large ES = 1.21–1.99; and extremely large ES > 2.0).

The software Statistica ver. 13.0 (Dell Inc., Tulsa, OK, USA) was used for all analyses, and a *p* level of 95% (*p* < 0.05) was applied.

## 5. Conclusions

According to the aim of this study, the obtained results demonstrated that different RMT modalities elicit different effects on respiratory muscles and small airway function. More precisely, the findings of this study indicated that EMs responded more significantly to forced breathing cycles with a short duration, resulting in an increase in MEP. Such results could be explained by the anatomical and physiological characteristics of EMs, including their higher percentage of fast-twitch type II fibers and a predominantly passive role in normal breathing. Hence, EMs are more responsive to short-duration and high-intensity training, which leads to rapid neuromuscular adaptations and sustained improvements in MEP. In contrast, IMs demonstrated non-significant changes in MIP. Additionally, IMs with predominant type I fibers require stimuli with a higher load and longer duration to induce physiological adaptations, which explains the limited changes in MIP.

Nevertheless, this study demonstrated sustained improvement in MEP after the 10-day washout period. This may be attributed to the fast-adaptive nature of EMs and their higher percentage of type II fibers. Due to increased motor unit recruitment and firing frequency, EMs tend to have rapid neuromuscular adaptation through stimuli. Moreover, because of the passive role of normal everyday breathing, the forced expiratory training phase provided a novel stimulus, enhancing voluntary activation and motor coordination. Such neuromuscular adaptations demonstrate a possible explanation for the persistent improvements after the 10-day washout period.

Additionally, the present study showed moderate improvement in MEF25% following the first training protocol and its regression to the baseline after the second protocol. This finding suggests that small airways have limited trainability due to the lack of smooth muscles and cartilage, with more likely structural than functional adaptation. In addition, enhancement in airflow could be established by temporary mechanical or effort-related factors rather than physiological adaptation. Furthermore, considering the high variability of the MEF25% parameter and difficult trainability, applied training protocols were insufficient to induce lasting effects on small airway function.

To conclude, properly designed RMT can selectively target specific respiratory muscles based on the structure and physiological demands of the training stimulus. Comparatively, RMT emphasizing short-duration and forced breathing cycles tends to be more effective for enhancing EM performance due to the fast-adaptive profile of type II fibers and neuromuscular mechanisms. Conversely, IMs, with predominantly type I fibers, require longer durations and higher loads to achieve significant improvements, which were not adequately demonstrated in the present study. Furthermore, even though a temporary enhancement in MEF25% flow was observed, the rapid return to baseline levels shows the limited training effects of the small airway. Importantly, this study highlights the importance of implementing comprehensive respiratory testing when applying RMT. Finally, different training protocols may provide substantial benefits for athletes by improving respiratory muscle performance, depending on their specific sports requirements.

Regardless of the results obtained, this study has several important limitations. First and foremost, the sample of participants is relatively small and insufficiently homogeneous in terms of age and sex. The results cannot be generalized and can only be applied to healthy, physically active adults. The conclusions about the effects of the RMT program in this study are not applicable to respiratory or cardiovascular patients. Additionally, although validated in previous studies, Airofit PRO™ is a device primarily intended for training, and as a method for measuring respiratory pressures it is not the most precise, which may introduce certain measurement errors. Furthermore, the four-week timeframe in which the program was conducted may be limiting in the context of potential structural adaptations of the respiratory muscles, which in most skeletal muscles typically occur after approximately eight weeks of consistent training. As a result, the relatively short intervention period in this study is more likely to have induced acute neuromuscular and functional changes rather than longer-term morphological adaptations. Moreover, the greatest limitation of this study is the absence of a control group of participants who could be measured at the same timepoints without undergoing the RMT program. Also, one of the limitations of the study is the absence of detailed information regarding inspiratory and expiratory resistance levels, making it unclear whether the observed changes in PEmax are a direct result of the prescribed resistance. Additionally, the lack of individualized resistance adjustments limits control over training intensity, which may affect the reproducibility and applicability of the findings in other groups.

In addition to these limitations, the study also has several strengths. First, the study implemented a higher training frequency and load compared to most previous research on similar samples. The sample consisted of participants with no prior experience in RMT training, so the observed changes can reasonably be expected to be solely related to the implemented training program. Also, this is one of the first studies to investigate the effects of RMT on the small airway.

Future studies should include larger and more diverse samples, longer intervention periods, more precise measurement tools, and appropriate control groups to improve the validity and generalizability of findings. Building on the strengths of this study, future research should further explore the effects of higher training loads and examine RMT impacts on the small airway across both healthy and clinical populations.

## Figures and Tables

**Table 1:** Differences between measurement timepoints in MIP and MEP.

Variable	Timepoints	Mean	SD	Timepoints		
				1	2	3
MIP (cmH_2_O)	1	142.63	24.85			
	2	139.75	31.68	0.57		
	3	147.13	27.58	0.66	0.89	
	4	135.63	25.71	0.19	0.46	0.38
MEP (cmH_2_O)	1	119.88	29.00			
	2	142.88	34.82	0.03 *		
	3	157.38	29.80	0.00 *	0.31	
	4	157.50	33.34	0.01 *	0.59	0.64

MIP, maximal inspiratory pressure; MEP, maximal expiratory pressure; SD, standard deviation; 1, baseline; 2, after first protocol; 3, after second protocol; 4, after washout; *, statistically significant value *p* < 0.005.

**Table 2:** Differences between measurement timepoints in PFT.

Variable	Timepoints	Mean	SD	Timepoints		
				1	2	3
FVC (L)	1	5.66	0.70			
	2	5.79	0.85	1.00		
	3	5.57	0.70	0.99	1.00	
	4	5.86	0.76	0.99	1.00	1.00
MEF75%	1	8.47	1.96			
	2	8.50	2.19	0.99		
	3	8.91	2.14	0.99	1.00	
	4	8.78	1.99	0.99	1.00	1.00
MEF50%	1	4.95	0.72			
	2	4.97	0.78	1.00		
	3	4.98	1.01	1.00	1.00	
	4	4.92	0.94	1.00	1.00	1.00
MEF25%	1	1.99	0.44			
	2	2.14	0.41	0.99		
	3	2.00	0.54	1.00	0.99	
	4	2.00	0.45	1.00	0.99	1.00
MVV (L)	1	180.27	34.57			
	2	194.06	45.95	0.12		
	3	199.82	42.71	0.16	0.87	
	4	202.09	42.54	0.16	0.87	1.00

FVC, forced vital capacity; MEF, maximal expiratory flow; MVV, maximal voluntary ventilation; SD, standard deviation; 1, baseline; 2, after first protocol; 3, after second protocol; 4, after washout.

## Data Availability

The original contributions presented in this study are included in the article. Further inquiries can be directed to the corresponding author.

## Abbreviations

The following abbreviations are used in this manuscript:

MIP	Inspiratory muscle pressure
MEP	Expiratory muscle pressure
FVC	Functional vital capacity
MVV	Maximal voluntary ventilation
MEF	Maximal expired frequency
